# Non-coding structural variants disrupting conserved *PITX2* enhancer loci in Axenfeld–Rieger syndrome

**DOI:** 10.1038/s41431-026-02086-x

**Published:** 2026-03-26

**Authors:** Lucas A. Mitchell, Joshua Schmidt, Emmanuelle Souzeau, Lachlan S. W. Knight, Giorgina Maxwell, Andrew Dubowsky, Ridia Lim, Edward Formaini, Matthew Welland, Cas Simons, Daniel G. MacArthur, Janey L. Wiggs, Jamie E. Craig, Owen M. Siggs

**Affiliations:** 1 Garvan Institute of Medical Research, Sydney, NSW Australia; 2 School of Clinical Medicine, Faculty of Medicine and Health, UNSW, Sydney, NSW Australia; 3 Department of Ophthalmology, Flinders University, Adelaide, SA Australia; 4 SA Pathology, Adelaide, SA Australia; 5 Sydney Eye Hospital, Sydney, NSW Australia; 6 Centre for Population Genomics, Garvan Institute of Medical Research, University of New South Wales, Sydney, NSW Australia; 7 Centre for Population Genomics, Murdoch Children’s Research Institute, Melbourne, VIC Australia; 8 Department of Ophthalmology, Harvard Medical School, Boston, MA USA

**Keywords:** Medical genomics, Genetics research

## Abstract

Axenfeld–Rieger Syndrome (ARS) is an autosomal dominant condition with both ocular and non-ocular manifestations. ARS is primarily caused by coding variants at the *PITX2* or *FOXC1* loci, yet many cases still remain undiagnosed. Here we used whole-genome sequencing to identify two families with non-coding structural variants associated with a typical presentation of *PITX2*-associated ARS: one family with a 450 kb deletion removing a series of conserved enhancer elements distal to *PITX2*, and the second family with a 12.54 Mb inversion displacing the *PITX2* gene from these same enhancer elements. Neither variant disrupted the *PITX2* gene itself, and therefore both were expected to reduce *PITX2* expression by disrupting its proximity or access to enhancer elements. *PITX2* enhancer-disrupting inversions are an emerging genetic mechanism for the development of ARS, which should be carefully considered in the context of ARS and other conditions without a conclusive genetic diagnosis.

## Introduction

Axenfeld–Rieger Syndrome (ARS) is a rare autosomal dominant condition with primarily ocular manifestations. Key ocular features of ARS include posterior embryotoxon, iridocorneal adhesions, corectopia and polycoria, with up to a 75% increased risk of developing glaucoma [1]. Non-ocular features of ARS include dental anomalies (hypodontia, microdontia), mild craniofacial dysmorphism, and umbilical anomalies (redundant periumbilical skin, umbilical hernia) [2–4].

ARS, as well as glaucoma associated with non-acquired ocular anomalies, is primarily caused by variation in *PITX2* or *FOXC1* [5]. Variants in these two genes account for approximately 70% of ARS cases, although it is unclear whether the remaining 30% are caused by undetected variants at these same loci,= or others [6]. Furthermore, the presence of microdontia/hypodontia and umbilical anomalies is highly specific for *PITX2*-associated ARS: in one case series these were observed in 91% and 94% of cases with *PITX2*-associated ARS (*n* = 59), as compared to 0% and 11% of *FOXC1*-associated ARS (*n* = 69) [6].

*PITX2* encodes a bicoid-like homeobox transcription factor that plays a vital role in regulating transcription during embryogenesis, particularly in the development of the eye and its anterior segment [7]. The locus encodes at least three isoforms, one of which (*PITX2*c) exhibits cardiomyocyte specificity, and putative gain-of-function missense variants unique to this isoform (p.Pro41Ser) have recently been associated with atrial fibrillation [8].

Most ARS-associated *PITX2* variants directly impact the canonical *PITX2* coding sequence, either via single-nucleotide variants, short indels, or partial and full-length gene deletions [6]. Upstream non-coding deletions of different sizes have also been described in at least seven unrelated ARS families [9–14]. These deletions all disrupt a series of conserved essential regulatory elements, with a common critical interval spanning three such elements (CE5-7, NC_000004.12:g.110926950-111115957) [9, 14].

Here, we describe an emerging genetic mechanism for ARS involving disruption of the *PITX2* enhancer locus. Through short-read whole genome sequencing (WGS) and structural variant calling, we identified two ARS families with non-coding structural variants adjacent to *PITX2*: in one family this involved a 450 kb non-coding deletion, and in a second unrelated family, a 12.54 Mb inversion. In both cases, the *PITX2* gene itself was not disrupted, only its adjacent enhancer elements, with the inversion variants causing a significant spatial displacement of *PITX2* from its enhancer.

## Methods

### Study participants

Patients and family members were recruited as part of the Australian and New Zealand Registry of Advanced Glaucoma [5]. Written informed consent was provided under protocols approved by the Southern Adelaide Clinical Human Research Ethics Committee (305–08), and adhering to the tenets of the revised Declaration of Helsinki.

### Exome and genome sequencing and analysis

DNA was prepared from venous blood samples, after temporary storage at −80 °C, using the QIAGEN DNeasy Blood and Tissue Kit (Hilden, Germany) and according to the manufacturer’s instructions. Whole exome sequencing was performed as previously described [15]. PCR-free WGS was performed by the Clinical Research Sequencing Platform at the Broad Institute. Library construction was performed using a KAPA HyperPrep kit, with libraries dual-barcoded and sequenced with 150 bp paired-end reads to a mean coverage of 30x. Alignment and calling of single-nucleotide variants (SNVs) and insertions/deletions (indels) were performed using the Illumina DRAGEN (Dynamic Read Analysis for GENomics) pipeline to reference genome hg38. Structural variants (SV) were called using the GATK-SV pipeline (https://github.com/broadinstitute/gatk-sv). All variants were analysed using the *seqr* platform and represented by HGVS nomenclature.

### SNP genotyping and CNV calling

Individuals from Family 1 were genotyped on either HumanOmniExpress-12-v1-0-K (I:2) or HumanCoreExome-24v1-0_A genotype (II:2) arrays. Each of the individuals reported here were members of larger batches of samples, with each batch assessed for quality using Illumina GenomeStudio. After export of ‘Genotype’, ‘Log R Ratio’ and ‘B Allele Frequency’, samples were assayed for CNV using *acne* (available at https://github.com/joshuamschmidt/acne). CNV calls were annotated and filtered using thresholds recommended in the PennCNV documentation.

## Results

We investigated two kindreds of European ancestry, both with a combination of ocular and systemic features typical of *PITX2*-associated ARS (Table 1), with phenotypic segregation suggesting autosomal dominant inheritance.

**Table 1:** Clinical features of recruited family members.

	Family 1 (450 kb deletion)		Family 2 (12.54 Mb inversion)		
Relationship to proband	I:2 (proband)	II:2 (proband’s daughter)	III:1 (proband)	III:2 (proband’s half brother)	II:2 (proband’s mother)
Sex	Female	Female	Male	Male	Female
Age at last examination (*y*)	67	47	6	3	27
BCVA	6/12;6/12	prosthesis/CF	6/48;HM	3/4.8;3/4.8	6/6;6/7.6
Highest IOP (mmHg)	36/42	./19	37/42	15/14	25/41
VCDR	0.9/0.95	./1.0	0.3;0.2	./.	0.2;0.2
Glaucoma [age at diagnosis, *y*]	Bilateral advanced glaucoma [24]	Bilateral advanced glaucoma [12]	Bilateral ocular hypertension [6]	./.	LE glaucoma [31]
CCT (μm)	452/417	./.	770/760	./.	582/587
Anterior segment	Abnormal iris collarettes, peripheral anterior synechiae	iris hypoplasia, posterior embryotoxon, corectopia, polycoria	Corneal opacity, peripheral anterior synechiae, band keratopathy	Iris hypoplasia, posterior embryotoxon, corectopia, polycoria	Iris hypoplasia, posterior embryotoxon, RE corectopia, LE polycoria
Ocular surgery	RE: trabeculectomy, PCIOL, Baerveldt tube LE: trabeculectomy, PCIOL, Gunderson flap	RE: prosthesis LE: trabeculectomy, corneal graft	RE: corneal grafts (2), cyclodiode LE: corneal graft, PCIOL, stent, Baerveldt tube	./.	./.
Systemic	Hypodontia, umbilical hernia	Hypodontia, umbilical hernia, stiff joints, double uterus, CAKUT	Redundant periumbilical skin, imperforate anus, hypodontia, microdontia, hearing loss	Redundant periumbilical skin, hypodontia, microdontia	Umbilical hernia, oligodontia, microdontia
Variant status	REF/ALT	REF/ALT	REF/ALT	REF/ALT	REF/ALT

Bilateral data is presented as RE/LE. Clinical data from individual III:1 (Family 1) were not available.

*y* years, *BCVA* best corrected visual acuity, *IOP* intraocular pressure, *VCDR* vertical cup-to-disc ratio, *CCT* central corneal thickness, *RE* right eye, *LE* left eye, *CF* count fingers, *HM* hand movements, *PCIOL* posterior chamber intraocular lens, *CAKUT* congenital anomalies of the kidney and urinary tract.

**Fig. 1:**
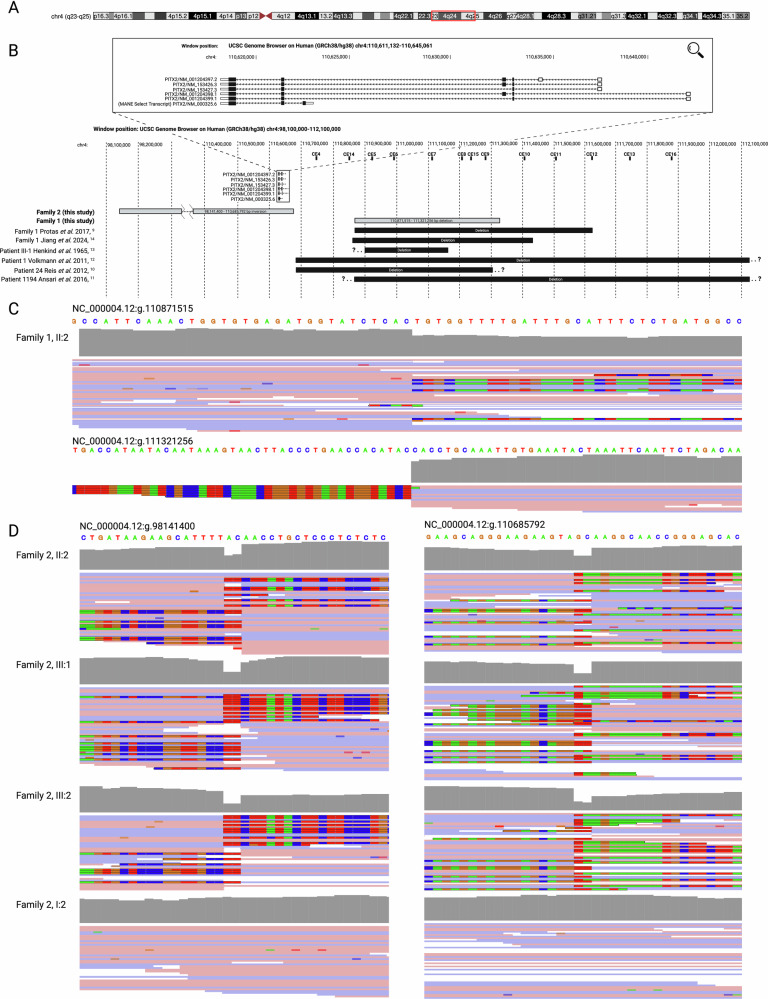
A 12.54 Mb inversion and 450 kb deletion at the *PITX2* locus. **A** Schematic of chromosome 4, highlighting the 12.54 Mb inverted region in red (UCSC Genome Browser, GRCh38/hg38, GENCODE V49). **B** Schematic of the *PITX2* locus and surrounding region (adapted from Protas et al. 2017 (9)), demonstrating the location of the 450 kb deletion (Family 1) and 12.54 Mb inversion (Family 2), along with previously reported *PITX2* enhancer deletions. Black boxes within the magnified transcripts indicate coding exons and white boxes indicate non-coding exons. Note that only the *PITX2* gene is shown here for simplicity: another 65 genes are within the inverted region indicated for Family 1. IGV read-level support for the **C** 450 kb deletion and **D** 12.54 Mb inversion. The absence of mapped reads at the proximal breakpoint in (C) is due to the presence of a common 4.89 kb deletion in trans (NC_000004.12:g.111316365-111321256, gnomAD v4.1.0 NFE AF = 0.1243).

The affected female proband of Family 1 (I:2) was initially subjected to genotyping array-based analysis, which revealed a potential heterozygous non-coding deletion distal to *PITX2*, inferred to be 482 kb in length (g.4:110890464_111372690del). Subsequent testing of the proband’s affected daughter (II:2) identified an overlapping heterozygous 477 kb deletion (g.4:110891306_111368288del). These were further resolved on WGS, which confirmed a shared heterozygous 450 kb deletion (NC_000004.12:g.110871515_111321256del), overlapping a series of 6 conserved non-coding elements (CE5-9 and CE15, Fig. 1B). The proband’s granddaughter (III:1) also reportedly had a clinical diagnosis of ARS, although did not undergo genetic testing. No other family members were known to have ARS, although the proband’s affected daughter (II:2) also had a double uterus and Congenital Anomalies of the Kidney and Urinary Tract (CAKUT) (Table 1), neither of which has previously been reported in the context of *PITX2*-associated ARS [2, 9, 11–14, 16] nor did we find a gene variant which could account for this on WGS.

Based on the clinical presentation of the male proband in Family 2 (III:1) (Table 1), in 2013 the complete coding sequence and intron-exon boundaries of *FOXC1* and *PITX2* were amplified by PCR and covered by capillary sequencing, and analysed for copy number variation by multiplex ligation-dependent probe amplification (MLPA), although neither assay revealed a likely genetic cause. The proband’s mother (II:2) was also referred for karyotype studies and array CGH (BlueGnome Cytochip oligo ISCA (4080-5) 8x60K array, 0.2 Mb resolution), including a detailed examination of the *FOXC1* and *PITX2* loci. Both assays were reported as normal.

In 2014, four members of Family 2 were then subjected to whole exome sequencing: the affected proband (III:1) and his affected half-brother (III:2), their affected mother (II:2), and their unaffected maternal grandmother (I:2). All other family members were reportedly unaffected. There were no rare and predicted deleterious SNVs or short indels in any genes associated with anterior segment dysgenesis, including missense, nonsense, and essential splice variants, although non-coding and structural variants were not systematically examined. Manual inspection of all coding exons of *FOXC1* and *PITX2* also did not reveal any significant copy number variants to suggest a deletion or duplication, nor did a genome-wide analysis of copy number variation based on exome or genotyping array data (Schmidt et al., in preparation).

Finally, in 2024, the same four members of Family 2 were subjected to whole genome sequencing. Again, close inspection of the *FOXC1* and *PITX2* loci did not reveal any rare SNVs or short indels with a predicted deleterious effect, nor did a careful manual inspection of coverage depth across both genes. A genome-wide structural variant callset was generated using the GATK-SV pipeline [17]: although this did not reveal any insertions or deletions at the *FOXC1* and *PITX2* loci, it did reveal a 12.54 Mb inversion on chromosome 4 (NC_000004.12:g.[98141400_98141401delinsTTCTTA;98141402_110685790inv;110685791_110685792del]), encompassing 66 genes including all coding exons of *PITX2* (Fig. 1). This inversion variant was present in a heterozygous state in all three affected individuals, and was absent from the unaffected maternal grandmother (I:2). This 12.54 Mb inversion was absent from the gnomAD SV v4.1.0 callset, and from a jointly-called dataset of 8920 alleles from a variety of rare disease patients and their family members.

Since *PITX2* was the most distal gene in the inverted region and adjacent to the distal breakpoint (TSS 43.7 kb proximal to the breakpoint) (Fig. 1B), we hypothesised that displacement from an enhancer locus could explain the observed phenotype. This distal breakpoint was found to be proximal to a series of conserved *PITX2* enhancer loci [9, 14], including those deleted in Family 1 and other previously reported families (Fig. 1B). The inversion variant would then be predicted to displace (and invert) the *PITX2* gene another 12.54 Mb proximal to these enhancers, leading to a predicted reduction in *PITX2* expression and haploinsufficient effect.

## Discussion

Here we describe two ARS families with non-coding structural variants: the first a 450 kb deletion, and the second a 12.54 Mb inversion. Both disrupt a series of conserved enhancer elements distal to the *PITX2* gene, three of which (CE5-7, NC_000004.12:g.110926950_111115957) form part of a shared critical interval across 7 previously reported families with enhancer deletions (Fig. 1B). In the case of the deletion observed in Family 1 these same elements were lost (in addition to CE8, CE9, and CE15), and in the case of the inversion in Family 2 these same elements were displaced from the *PITX2* gene body.

In light of recurrent non-coding deletions at the *PITX2* locus, the haploinsufficient nature of *PITX2*-associated ARS, and the highly specific phenotype observed here, the most likely consequence of the Family 2 inversion appears to be a loss of *PITX2* expression due to the displacement of the *PITX2* gene body from its enhancer loci, although this is yet to be confirmed experimentally. A similar mechanism may also be at play in other ARS cases associated with balanced translocations (*t*(4;16) and *t*(4;11)) and a pericentric inversion with an undefined breakpoint spanning *PITX2* and its enhancer [16, 18]. Although specific details are yet to be published, a similar mechanism has been suggested for two separate inversions at the *PITX2* locus [19].

This mechanism has also been suggested in other developmental eye disorders. For example, a de novo inversion in a girl with congenital aniridia was found to separate *PAX6* from its enhancers and was associated with reduced *PAX6* RNA expression in patient-derived cell lines [20]. A similar study reported a case of a boy with ocular, skeletal and neurological features associated with a de novo inversion 100 kb downstream of *FOXC1*, associated with allele-specific loss of *FOXC1* expression in patient-derived conjunctival cells [21]. These cases collectively highlight that although some structural variants may not disrupt the gene sequence itself, they may still alter gene expression by disrupting the relative position of adjacent regulatory elements [19–21].

In summary, enhancer-disrupting deletions and intergenic inversions represent an emerging genetic mechanism for the development of *PITX2*-associated ARS, which should be carefully considered in any ARS families, or indeed any suspected monogenic condition, where a causal variant has not been confidently assigned.

## Data Availability

Any additional data not included in the manuscript is available from the corresponding author upon reasonable request. The *PITX2* 12.54 Mb inversion variant has been submitted to ClinVar (accession: SCV007328912).
